# Update of Takotsubo cardiomyopathy: Present experience and outlook for the future

**DOI:** 10.1016/j.ijcha.2022.100990

**Published:** 2022-03-07

**Authors:** Anastasiia V. Bairashevskaia, Sofiya Y. Belogubova, Mikhail R. Kondratiuk, Daria S. Rudnova, Susanna S. Sologova, Olga I. Tereshkina, Esma I. Avakyan

**Affiliations:** a Department of Paediatrics, Institute of Child’s Health, Sechenov First Moscow State Medical University (Sechenov University), 119435 Moscow, Russia; b Department of Faculty Therapy, Sechenov First Moscow State Medical University (Sechenov University), 119991 Moscow, Russia; c AMEE International Networking Centre, Sechenov First Moscow State Medical University (Sechenov University), 123242 Moscow, Russia; d International School “Medicine of the Future”, Sechenov First Moscow State Medical University (Sechenov University), 119991 Moscow, Russia; e Department of Pharmacology, Institute of Pharmacy, Sechenov First Moscow State Medical University (Sechenov University), 119571 Moscow, Russia

**Keywords:** Takotsubo, Cardiomyopathy, Risk factors, Diagnostics, Complication, Treatment

## Abstract

Takotsubo cardiomyopathy (TTS) has become a recognised clinical entity since the Japanese scientist Sato first described it in 1990.

Despite an increasing number of confirmed cases, especially during the COVID-19 pandemic, its pathophysiology remains incompletely understood, and decision-making differs in the diagnosis and treatment. In addition, it is not evident whether a significant increase in TTS is due to better understanding among practitioners and widespread access to coronary angiography, or if it is a reflection of an actual increase in incidence.

We analysed a series of international research studies from 1990 to 2021. Beyond epidemiology and clinical presentation, we evaluated and summarised fundamental knowledge about various predisposing factors, with particular attention to the iatrogenic impact of certain drugs, namely antidepressants, chemotherapy, and antiarrhythmics.

Furthermore, we highlighted the main pathophysiological theories to date. In addition, based on published studies and clinical cases, we investigated the role of numerous diagnostic approaches in the differential diagnosis of TTS and identified predictors of TTS complications, such as cardiogenic shock, ventricular fibrillation, and left ventricular thrombi. Accordingly, we sought to propose a diagnostic algorithm and further treatment management of TTS under the presence of possible complications to help practitioners make more informed decisions, as the initial presentation continues to pose a challenge due to its close similarity to acute coronary syndrome with ST-elevation.

In conclusion, this article examines Takotsubo cardiomyopathy from different perspectives and, along with future systematic reviews and *meta*-analyses, can be of particular interest to practising cardiologists and researchers in developing clinical guidelines.

## 1 Introduction

Takotsubo syndrome (TTS), also known as stress-induced cardiomyopathy or broken heart syndrome, is reversible cardiomyopathy characterised by acute transient left ventricular (LV) dysfunction and is mostly associated with LV apical distension visualised during systole [1].

It was initially thought to be a benign condition, but it is now recognised to be associated with severe complications such as life-threatening ventricular arrhythmias and cardiogenic shock. In addition, the incidence of Takotsubo syndrome has been increasing [2], [3], and the process appears to be multifactorial. Recognition and increased awareness, along with improved technologies, have helped the diagnostic evaluation of TTS. However, there are still no generally established clinical guidelines for diagnosing, treating, and preventing TTS.

This article aims to review the current situation of TTS, briefly summarize pathophysiological mechanisms, discuss various risk factors, propose a diagnostic algorithm and different treatment strategies of the patients with stress-induced cardiomyopathy, and highlight future challenges.

## 2 Epidemiology

The exact prevalence of TTS is uncertain because it can be misdiagnosed and hardly distinguished from acute coronary syndrome (ACS) without advanced diagnostic tools. In the United States Nationwide Inpatient Sample discharge records the reported prevalence of TTS was 5.2/100,000 for females and 0.6/100,000 for males, thus accounting for 0,02% of hospitalizations [4], [5]. It has also been reported that the combined annual incidence of principal and secondary TTS hospitalizations increased from 5.7/100,000 person-years in 2007 to 17.4/100,000 in 2012 [2], [3]. Coronary artery disease (CAD) was frequently present in patients with TTS, and according to the HORIZONS-AMI trial in 2014, TTS was identified in 2.1% of female ACS patients and 0.5% of all ACS patients [6].

Important to note a considerable increase in the incidence of TTS during the COVID-19 pandemic. As reported in the retrospective large cohort Cleveland Clinic study, the proportion of TTS cases among all ACS patients before and during the pandemic was 1,8% and 7,8%, respectively [7].

The multicenter GEIST (German Italian Stress Cardiomyopathy) Registry analysed the recurrence rate of TTS which accounted for 4.0% among 749 consecutive patients with TTS and a median follow-up of 830 days. Relatively younger age and the presence of cardiovascular risk factors such as arterial hypertension were significantly higher in patients with recurrence episodes [8].

According to the International Expert Consensus Document on Takotsubo Syndrome, about 90% of TTS patients are women aged 67–70 [5]. Although women are more predisposed to stress-induced cardiomyopathy caused by emotional triggers, TTS tends to develop more often among male patients after a stressful physical triggering event with a higher mortality rate because of underlying critical illnesses [9], [10].

Based on the analyses of various types of studies, Okura et al. have demonstrated that emotional stress was strongly related with TTS development in about 20–39%, while physical stress was seen in 35–55% of all cases [11].

Moreover, TTS has been described in children, including premature neonates [5]. A retrospective analysis of the Healthcare Cost and Utilisation 2012 and 2016 Kids' Inpatient Database revealed TTS in 3.1 young patients per 100,000 discharges, aged 12–20. TTS occurrence was mainly associated with psychiatric illnesses, substance use disorder, and sepsis. Currently, there is no specific guideline regarding the diagnosis and management of TTS in children. Although it is an uncommon condition among children, it is essential to be aware of its association with other comorbidities [12].

## 3 Predisposing factors

The main predisposing factor includes female sex in a postmenopausal period. Some researchers linked the predominance of women to the absence of cardioprotective effects of androgens, which are observed in men. In particular, testosterone inhibits oxidative stress by activating cardiomyocyte antioxidant enzymes via androgen receptors [13] or by converting to estradiol [14].

In addition to hormonal changes, genetic and psychogenic factors, mental disorders, and other comorbidities influence TTS development (Table 1).

**Table 1:** Predisposing factors.

Predisposing factors	Description	Authors, year of paper
**Genetic factors**	People carrying the T allele at the rs2234693 locus of the ESR1 and rs1271572 locus of the ESR2 gene have a higher risk of TTS development.	Pizzino et al., 2017 [185]
	The Gln27Glu substitution at the rs1042714 within the adrenergic receptor B2 was observed more frequently in healthy controls than in TTS patients.	Vriz et al., 2011 [186]
	The Arg389Gly substitution at the rs1801253 of the adrenergic receptor B1 was more frequently found in TTS patients.	Vriz et al., 2011 [186]
	There is no significant difference between TTS and healthy controls in the Arg389Gly substitution at the rs1801253 of the adrenergic receptor B1.	Figtree et al., 2013 [187] Mattsson et al., 2018 [188]
	Each patient carried predicted deleterious variants affecting known cardiomyopathy genes.	Kalani et al., 2016 [189]
	A patient with TTS was detected with a heterozygous mutation in exon 9 of the TTN gene: c.1489G > T (p. E497X).	Keller et al., 2018 [190]
	APOE, MFGE8, ALB, APOB, SAA1, A2M, and C3 genes were classified as key genes of TTS, could be helpful either as diagnostic biomarkers or molecular targets for the treatment of TTS.	Pan et al., 2020 [191]
**Postmenopausal women/ hormonal changes**	Oestrogen may be considered a relative protective hormone; its decline may predispose to TTS development.	Pelliccia et al., 2017 [56]
	Oestradiol has been reported to be protective against catecholamine surge; reducing oestrogen levels may increase the risk of acquired long QT syndrome in TTS.	El-Battrawy et al., 2018 [179]
	Oestradiol deficiency was not identified as a risk factor for TTS.	Möller et al., 2018 [156]
	Vitamin D deficiency was more prevalent in patients with TTS. Probably, lack of Vitamin D leads to suboptimal myocardial reserve and worse hemodynamics in affected female patients.	Dande et al., 2013 [192]
**Psychogenic factors/ psychiatric disorders**	Higher rates of neurologic or psychiatric disorders (55.8% vs. 25.7%) were seen in patients with TTS than those with acute coronary syndrome.	Templin et al., 2015 [75]
	Common psychiatric disorders among patients with TTS included depression (n = 98; 39%), anxiety (n = 44, 17%), alcohol exposure (n = 35, 14%), suicidal thoughts (n = 30, 12%), and severe mental conditions (n = 25, 10%). Psychiatric illnesses were reported to be triggers for TTS development in 61% cases.	Carroll et al., 2020 [15]
	Among psychiatric events, long-lasting psychological distress and anxiety appeared to play a significant role in TTS predisposition.	Galli et al., 2019 [193]
	Anxiety disorders were reported to have a higher predisposition to TTS compared to myocardial infarction. Conversely, the prevalence of depression was the same between TTS and myocardial infarction groups. Role of personality appeared to not influence TTS development compared to myocardial infarction.	Salmoirago-Blotcher et al., 2016 [194]
**Comorbidities**	Concomitant diabetes mellitus in TTS patients is associated with an increased risk for worst outcomes.	Abe et al., 2020 [182]
	Underlying critical illness contributed most to mortality. Male patients, who have underlying critical illness more frequently, had a significantly increased mortality rate than female patients.	Brinjikji et al., 2012 [10]
	Underlying atrial fibrillation in TTS patients has a significantly higher all‐cause mortality rate than patients without atrial fibrillation (OR = 2.19, 95% CI: 1.57–3.06, p < 0.001).	Prasitlumkum et al., 2018 [195]
	TTS triggered by physical stress (i.e., asthma, surgery, trauma, drugs) had worse short and long-term outcomes, higher mortality rates, recurrences, and readmissions.	Núñez-Gil et al., 2015 [73]

Table summarising main predisposing factors in the TTS development.

TTS — Takotsubo cardiomyopathy, ESR1 – Estrogen Receptor 1, ESR2 – Estrogen Receptor 2.

Of note, despite the clear presence of mental disorder as a clinical substrate and trigger of TTS, psychiatric treatment during hospitalisation was provided in only 32% of cases. Furthermore, psychiatric disorders were not frequently managed during follow-up [15].

The effect of race on the incidence and prognosis of TTS has also been studied. For example, small-sampled research has demonstrated an increased in-hospital mortality rate among patients of African-American (AA) descent [16], [17]; however, a large population-based study has not reported racial differences for mortality [10]. Similarly, after a fully adjusted analysis, a recent study involving 97,650 patients, which included differences in age, comorbidities, and socioeconomic factors, has demonstrated that the African-American race was not associated with the worse in-hospital outcome than Caucasians.

In 2020, Jabri et al. carried out a cohort study that showed a significant increase in the number of confirmed TTS associated with the COVID-19 pandemic [7].

In addition, based on retrospective case series, complications and mortality rates in secondary TTS associated with COVID-19 are higher in older patients (>70 years), patients with lower blood pressure (<110 mm Hg), lower left ventricular ejection fraction (LVEF < 45%), presence of right ventricle (RV) involvement, and mitral regurgitation [18].

## 4 Drugs as risk factors for TTS

### 4.1 Antidepressants

Despite the high prevalence of depression and other psychiatric disorders among patients with TTS, certain antidepressants may still contribute to TTS development. Therefore, a series of clinical cases were analysed to identify the drugs contributing to TTS (Table 2). According to this data review, the TTS risk is highest in serotonin-norepinephrine reuptake inhibitors use, i.e., venlafaxine, desvenlafaxine, duloxetine, milnacipran. In addition, the use of selective serotonin reuptake inhibitors (SSRIs) like fluoxetine or selective norepinephrine reuptake inhibitors (SNRIs) as atomoxetine, or tetracyclic antidepressants such as maprotiline, may also be associated with TTS development.

**Table 2:** Antidepressants as risk factors for TTS development.

Drugs	Drugs for the treatment	Number of patients	Author, year
**Venlafaxine, Desvenlafaxine**	—	6	Neil et al., 2012 [196]
**Desvenlafaxine**	Bisoprolol, perindopril (combination therapy of β-blockers and ACE inhibitors)	1 (F)	Gurunathan, 2018 [197]
**Duloxetine**	Carvedilol (the patient used it before the TTS), the addition of ACE inhibitors	1 (F)	Selke et al., 2011 [198]
**Duloxetine**	—	1 (F)	Rotondi et al., 2011 [199]
**Maprotiline**	Bisoprolol, verapamil, warfarin (already used by the patient), therapy with medosalam, cyproheptadine	1 (F)	Sasaki et al., 2012 [200]
**Milnacipran**	Enalapril, carvedilol, ACE inhibitors (combination therapy of β-blockers and ACE inhibitors)	1 (F)	Forman et al., 2011 [201]
**Venlafaxine**	ILE, ECLS and CytoSorb®	1 (F)	Schroeder et al., 2017 [202]
**Venlafaxine**	Aspirin, enalapril, metoprolol (combination therapy of β-blockers and ACE inhibitors)	1 (F)	Vasudev et al., 2016 [203]
**Venlafaxine**	Combination therapy of β-blockers and ACE inhibitors	1 (F)	Christoph et al., 2010 [204]
**Venlafaxine**	Ramipril, carvedilol, diuretics	1 (F)	Elikowski et al., 2019 [205]
**Atomoxetine, Fluoxetine**	—	1 (F)	Naguy, 2016 [206]
**Fluoxetine**	Heparin, clopidogrel, a chewable aspirin, atorvastatin, lisinopril (only ACE inhibitors)	1 (F)	Conrad et al., 2016 [207]
**Atomoxetine, Fluoxetine**	—	2	Verduin, 2016 [208]
**Fluoxetine**	Metoprolol, ramipril (combination therapy of β-blockers and ACE inhibitors)	1 (F)	Prasad, 2009 [209]

Table summarising studies on the role of antidepressants in the TTS development.

ACE inhibitor — Angiotensin-converting enzyme inhibitor, TTS — Takotsubo cardiomyopathy, ILE — Intravenous Lipid Emulsion, ECLS — Extracorporeal Life Support.

SNRIs block the capture of neurotransmitters in the presynaptic membrane, which increases the level of noradrenaline. This process leads to activation of the sympathoadrenal system. The mechanism of action of SSRIs on the development of TTS is not fully understood, although overdoses and drug interactions have been reported. In 2002, a study showed that only fluoxetine increased norepinephrine concentrations among SSRIs, contributing to the development of TTS. In addition, it was found that its extracellular concentrations in the prefrontal cortex at a dose that increased extracellular monoamines (in this case, serotonin) were 242 nM, enough to block 5-HT(2C) receptors, which is a possible mechanism for fluoxetine-mediated catecholamine increase [19].

Therefore, before starting antidepressant therapy, physicians are advised to obtain a comprehensive medical and family history, paying attention to sudden cardiac death and heart failure. Furthermore, according to the analysed data, tricyclic antidepressants, SSRIs, and SNRIs should not be prescribed in patients with heart disease due to a high risk of drug-drug interaction resulting in an increased sensitivity of cardiomyocytes to catecholamines. For that reason, in case of drug-induced TTS, these drugs should be withdrawn and, if possible, replaced with other medications (for example, paroxetine, sertraline, or plant-based drugs such as valerian or motherwort).

### 4.2 Chemotherapy

Carbone A. et al. have found that TTS development was associated with a broad spectrum of antitumoral drugs used in various cancers. For example, treatment with 5-fluorouracil, capecitabine, trastuzumab, and immune checkpoint inhibitors was found in 36.5%, 9.7%, 9.7%, 9.7% of TTS cases, respectively [20]. The exact mechanism of chemotherapy-induced cardiotoxicity of 5-fluorouracil and capecitabine is not fully known. However, coronary spasm, thromboxane A2 activation, and electrolyte imbalances are the most likely effects [21], [22], [23], [24]. Trastuzumab prompts extracellular signal-regulated kinase activation, leading to increased regulation of the mammalian target of rapamycin (mTOR) [25]. Activated mTOR phosphorylates Unc51-like kinase that inhibits autophagy, thereby disrupting the ability of cardiomyocytes to utilise toxic cellular substrates, leading to direct myocardial damage [26]. Bevacizumab disrupts cardiomyocyte survival via vascular endothelial growth factor (VEGF) signalling inhibition [27]. It is hypothesised that inhibition of VEGF reduces nitric oxide production in the endothelium, leading to vasoconstriction, increased peripheral vascular resistance, and blood pressure. Reduced nitric oxide levels contribute to the expression of plasminogen activator inhibitor 1, leading to a further increase in blood pressure and cardiac stress [28]. The cardiotoxic effect of immune checkpoint inhibitors is still unknown.

### 4.3 Antiarrhythmic drugs

In addition, based on a series of clinical cases, antiarrhythmic drugs have been identified as risk factors for TTS development, especially drugs such as flecainide, sotalol, amiodarone, lidocaine, and xylocaine (Table 3). Flecainide leads to marked inhibition of the upstroke of the cardiac action potential, delayed inactivation of the slow sodium channel, and blocks the delayed rectifier potassium channels (IKr3), resulting in a prolonged QT interval, potentially preceding a TTS [29]. Amiodarone by blocking potassium rectifier currents leads to an increase in the action potential duration and prolongation of the effective refractory period in cardiomyocytes and therefore can prolong QTc interval. Amiodarone also interferes with beta-adrenergic receptors (e.g., beta-1), calcium and sodium channels, which can induce arrhythmias and worsen the course of TTS [30].

**Table 3:** Potential drugs as risk factors for TTS development.

**Antineoplastic drugs**						
**5-Fluorouracil**	Age, sex	Evaluation criteria	Duration of observation	Relapse /complications	Additions	Reference
	48, M	Chest pain ECG: abnormal ST Echo-CG: severe LV dysfunction, hypokinesia in the apical and median segments, LVEF 15%	5 months and 3 weeks	Relapse after 1.5 months with cardiac arrest and acute HF	oxaliplatin, calcium folinate	Basselin C et al., 2011 [210]
	58, F	Increased serum troponin T levels by 0.39 mg / L, creatine kinase 156U / L, Echo-CG: severe diffuse hypokinesis of the LV, LVEF 15%; ECG: non- specific lateral ST changes and poor R wave progression	3 months	No	oxaliplatin, folinic acid	Stewart T et al., 2010 [211]
	48, M	Tachycardia, troponin-I 2,87 ng / ml ECG: negative T at V4,5; Echo-CG: severe LV dysfunction with hyperkinesis of the mid-apical segments and hyperkinesis of the basal segments, LVEF 15%	13.5 months	Ventricular fibrillation, death	docetaxel, cisplatin, irinotecan, folinic acid	Ozturk M et al., 2013 [21]
	35, M	Echo-CG: severe LV dysfunction, LVEF 19%	6 months	pulmonary oedema	oxaliplatin, folinic acid	Saif M et al., 2016 [212]
	42, F	ECG: elevation of the ST segment in the anterior leads and depression of the ST segment in the inferolateral leads Echo-CG: LVEF 17%, general hypokinesis with apical akinesia	3 months	LV thrombus	No	Kumar D et al., 2021 [213]
Capecitabine	80, F	ECG: hyperacute T waves in the anterior precordial leads; Ventriculography: apical ballooning with a hyperdynamic base; Echo-CG: LVEF 55–60%, apical akinesia	6 weeks	No	clonazepam, atorvastatin, meclizine	Bhardwaj P et al., 2019 [214]
	55, M	Troponin T 89 ng / l ECG: elevation of the ST segment by 1–2 mm in V2-6 and about 1 mm in -aVR, II and aVF Echo-CG: LVEF 15–20%, severe LV hypokinesis;	1 week	No	No	Y-Hassan S et al., 2013 [215]
	47, M	Serum troponin I increased to 0.19 ng/ml; ECG: ST-segment elevations in inferolateral leads I, II, aVL and V6, and ST depression and T wave inversion in leads V1-V2; Ventriculography: anomaly of movement of the anterior apical wall with LVEF 35%; Echo-CG: general hypokinesia	6 weeks	No	doxorubicin, paclitaxel, vinorelbine, tamoxifen, and anastrozole	Qasem A et al., 2016 [216]
Gemcitabine	65, F	TnT 111 ng / ml; ECG: T wave inversion in precordial leads; Echo-CG: LV apical balloon, LVEF 25–30%	Still now	No	vinorelbine	Ozbay B et al., 2021 [217]
	62, F	troponin I − 9552 ng / l, BNP − 175 pg/ml, NT-proBNP − 2691 pg/ml ECG: inversion of T waves in leads above the anterior and lateral walls; Ventriculography: akinesia of the apex, apical and middle segment of the anterior wall, LVEF 38%;	4 days	No	cisplatin	Zalewska-Adamiec M et al., 2021 [218]
Trastuzumab	65, M	Troponin I 0.219 ng / ml, creatinine kinase (CC) 104 U / l; Fever, shortness of breath, chest pain; ECG: negative T waves in V2 and ST-segment elevation in V3 - V6, apical akineдsia and LV hypokinesia with an apical balloon, LVEF 33,1%	23 days	RDS, death (as a result of carcinoma)	oxaliplatin	Matsumoto T et al., 2019 [219]
	40, F	Troponin 2.64 μg / L;HR 105 btm/min, arterial hypotension (98/50 mm Hg); Echo-CG: LVEF 45%; left ventriculography: severe hypokinesia of the middle LV segment	1 year and 8 months	No	carboplatin, docetaxel	Burgy M et al., 2014 [220]
	50, F	Chest pain, sweating, nausea, increased troponin T levels (0.15 pkg/ml); ECG: negative T waves in leads V1-3, I, aVL.	6 weeks	No	carboplatin, docetaxel	Khanji M et al., 2013 [221]
**Antiarrhythmic drugs**						
**Flecainide**	48, M	ECG: initially QT prolongation (QTc 624 ms), then negative T in leads I, aVL, V2 - V6	A few months	No	Emotional stress before hospitalisation	Bodziock G et al., 2019 [222]
**Sotalol**	82, M	Echo-CG: LV hypokinesis with LVEF 25%; ECG: QT prolongation with abnormal T waves	3 weeks	No	Emotional stress before hospitalisation	Friedman P et al., 2010 [223]
**Amiodarone**	79, F	Echo-CG: akinesia of the apical and middle segments with hypokinesia in the basal segments; ventriculography: apical dyskinesia, with the apical balloon and preserved LVEF	3 months	New episode of ischemic colitis with bowel perforation, generalised sepsis, and death	Hyperthyroidism type I	Capel I et al., 2017 [224]
**Lidocaine**	36, F	Palpitations, nausea, troponin-T 1.03 ng / ml; NT-pro-BNP 717 pg / ml; ECG: ST-segment elevation and negative T wave in aVL; Ultrasound: hypokinesia of the basal segments and hyperkinesis of the apex with moderate mitral regurgitation	1 month	No	No	Tomcsányi J et al., 2008 [225]
	14, F	Hypotension (89/56 mm Hg); troponin I 2.42 ng / ml; proBNP 8284 pg / ml; total creatine kinase 217UI / l; Echo-CG: dilated LV with hypokinesia of the middle and basal segments, LVEF < 30%; ECG: non-specific ST wave abnormalities in V4-V5;	9 days	No	Propofol, Atropine	Faleiro Oliveira J et al., 2016 [226]
**Xylocaine**	18, F	HR 140 bpm; BP 86/52 mm Hg.; increased troponin and creatine kinase ECG: non-specific ST wave abnormalities; Echo-CG: LV apical hypokinesis with anomalies of the anterior wall movement, LVEF 15–20%	6 days	No	Epinephrine	Glamore M et al., 2012 [227]

Table summarising studies on various drugs as potential risk factors for TTS.

M — Male, F — Female, ECG — Electrocardiography, Echo-CG — Echocardiography, LVEF — Left ventricular ejection fraction, LV — Left ventricle, TnT — Troponin-T, BNP — Brain natriuretic peptide, NT-proBNP — N-terminal-pro brain natriuretic peptide, RDS — Respiratory distress syndrome, HR — Heart rate, BP — Blood pressure.

Although it was difficult to distinguish which factor may have played a significant role in TTS development, a detailed cardiovascular assessment, prior to initiating antiarrhythmic therapy, and routine ECG screening for QT interval prolongation will minimise the risks of life-threatening arrhythmia resulting in the development of “stunned” myocardium with the features of apical ballooning syndrome in susceptible patients.

## 5 Pathogenesis

The pathogenesis of TTS remains elusive and unclear. However, the main pathogenic hypotheses include catecholamine surge, switching in G-coupled proteins, systemic inflammation, endothelial dysfunction, coronary vasospasm and excessive activation of the sympathetic nervous system (SNS) (Table 4).

**Table 4:** Pathogenesis.

Pathogenetic pathways	Description	Authors, year of paper
**Excessive activation of the sympathetic nervous system**	Sympathetic nervous system activation in response to stress leads to increased catecholamine release via the hypothalamic–pituitaryadrenal axis.	Wang et al., 2020 [31]
**The role of the limbic system**	The presence of dysregulated neural networks in several stress-associated limbic brain regions such as the amygdala, insula, anterior cingulate cortex, prefrontal cortex, and hippocampus was highlighted among patients with TTS.	Suzuki et al., 2014 [35] Radfar et al., 2021 [36] Hiestand et al., 2018 [37] Pereira et al., 2016 [38]
**Catecholamine effects**	A higher level of circulating catecholamines was found in patients with stress-associated TTS than patients with myocardial infarction.	Wittstein et al., 2005 [47]
	Elevated concentrations of norepinephrine in coronary sinuses were found in patients with TTS.	Kume et al., 2008 [46]
	No elevated plasma catecholamine concentrations were detected in TTS patients.	Madhavan et al., 2009 [48]
	Intravenous administration of adrenaline or beta-receptor agonists can cause TTS symptoms.	Abraham et al., 2009 [228]
	Nearly 68% of drug-induced TTS cases were associated with catecholamine stimulation.	Kido et al., 2017 [49]
	Catecholamines can affect sarcoplasmic-Ca2 + -ATPase (SERCA2a) by stimulating β-adrenoreceptors and reducing sarcolipin concentrations.	Nef et al., 2009 [45] Nef et al., 2010 [44]
	The effect of catecholamines on β2-adrenoreceptors leads to activation of nitric oxide synthase, which stimulates nitric oxide synthesis. This results in the formation of peroxynitrite, which damages DNA and stimulates the DNA repairing enzyme poly(ADP-ribose) polymerase to be released.	Dawson et al., 2015 [52]
	Activation of adrenoreceptors enhances the differentiation of cardiomyocytes from iPSC-CMs. The main pathway of iPSC-CMs differentiation regulation is mediated through the alpha1-receptors.	Li et al., 2017 [55]
**Switching in G-coupled proteins**	Switching epinephrine affinity from beta2-adrenoreceptors-Gs at low epinephrine concentration to Gi at high epinephrine concentration leads to acute apical cardiac depression in a Takotsubo rat model. This epinephrine-affinity switching seems to be cardioprotective and decrease catecholamine-induced myocardial toxicity during acute stress.	Paur et al., 2012 [42]
	In the basal myocardium, norepinephrine contributes to hypercontractility due to the high density of sympathetic nerve endings. Conversely, the apical myocardium has an increased density of beta-adrenoreceptors. Thus, high free epinephrine leads to switching to beta2-receptors-Gi followed by apical myocardium dilatation.	Yoshikawa, 2015 [229]
**Systemic inflammation**	TTS is associated with a low-grade chronic inflammatory state. It consists of a myocardial macrophage inflammatory infiltrate and an increase in systemic proinflammatory cytokines (i.e., serum interleukin-6, chemokine ligand 1, and classic CD14++CD16-).	Scally et al., 2019 [62]
**Endothelial dysfunction**	Endothelial dysfunction is significantly increased in TTS patients compared to healthy controls.	Naegele et al., 2016 [57]
	TTS is significantly associated with migraine and the Raynaud phenomenon, which suggest a vasomotor dysfunction pathway in the TTS pathogenesis.	Scantlebury et al., 2013 [230]

Table summarising main pathogenetic pathways of TTS.

TTS — Takotsubo cardiomyopathy, DNA — Deoxyribonucleic acid, ADP — Adenosine diphosphate, iPSC-CMs — Induced pluripotent stem-cell-derived cardiomyocytes.

One of the most popular theories of pathogenesis is activation of the sympathetic nervous system (SNS) in response to stress, which results in over-release of catecholamines [31]. Increased SNS activity was first demonstrated in a study by Akashi et al. who used 123I-metaiodobenzylguanidine (MIBG) myocardial scintigraphy [32]. Ten years later, Vaccaro et al. confirmed the presence of increased SNS activity in patients with TTS, using the evaluation of spontaneous baroreflex control of sympathetic activity and microneurography [33]. Although the part of the sympathetic nervous system is heavily discussed, the role of the parasympathetic nervous system (PNS) often has been neglected. Norcliffe-Kaufmann et al. assessed sympathetic and parasympathetic activity using baroreflex, cognitive, and emotional stimulation; their results showed a reduction in parasympathetic modulation of heart rate in patients with TTS [34]. Some recent neuroimaging studies provide strong evidence that alterations in the central autonomic network (CEN) are found to be present in patients with TTS. Other studies have demonstrated the presence of dysregulated neural networks in several stress-associated limbic brain regions such as the amygdala, insula, anterior cingulate cortex, prefrontal cortex, and hippocampus among patients with TTS [35], [36], [37], [38]. Although alterations were found, it is still unclear whether they are predisposing or causal factors for TTS development. It was also observed that when the spinal cord was transected at the C7-Th1 level in rats, followed by adrenaline administration, myocardial damage was significantly reduced, confirming the role of the central nervous system in the pathogenesis of TTS [39], [40].

There are two phases in the catecholamine hypothesis. The first phase is characterised by a massive release of catecholamines in response to stress. The second phase reflects cardiovascular reaction to a rapid increase in plasma level of catecholamines. As a result, apical dysfunction, myocardial stunning, and paradoxical vasodilation develop, culminating in a decrease in cardiac output, systemic hypotension, and acute heart failure [41]. These changes are considered as a cardioprotective mechanism that is caused by beta-receptors Gs-Gi switching [42]. Although Gi-pathway inhibition was associated with decreased cardiac akinesia in a rat model, it significantly increased mortality [43]. With reference to these findings, TTS patients may benefit from beta-blockers because they bind to and inhibit the Gs-protein metabolic pathway in cardiomyocytes via beta-adrenoreceptors downregulation. In addition, it has been shown that catecholamines can affect sarcoplasmic-Ca2 + -ATPase (SERCA2a) by stimulating β-adrenoreceptors and reducing sarcolipin concentrations. At the same time, phospholamban is dephosphorylated and decreases the affinity of Ca2 + -ATPase for calcium. A subsequent increase in phospholamban/SERCA2a ratio leads to contractile dysfunction [44], [45].

Even though a high level of circulating catecholamines was found in patients with stress-associated TTS, the role of catecholamine effects is still not entirely understood [46], [47]. Contradicting research demonstrated no differences in elevated plasma catecholamine concentrations between TTS and STEMI patients, which the following factors can explain: first of all, TTS patients are a heterogeneous group with variable mechanisms of systolic dysfunction; in addition, laboratory data discrepancies can be attributed to the difficulty in measuring catecholamines, differences in assays, and cut-off values for what is considered normal [48]. Conversely, the systematic review of 157 cases demonstrated that 68.2% of drug-induced TTS cases were associated with catecholamine stimulation, while 8.9% were due to chemotherapy-induced coronary vasospasm [49].

It has been demonstrated that the effect of catecholamines on β2-adrenoreceptors leads to activation of nitric oxide synthase (NOS), which stimulates nitric oxide (NO) synthesis [50]. In addition, the synthesis of reactive oxygen species is increased in patients with TTS. This results in the formation of peroxynitrite - a product of the reaction of NO and reactive oxygen species superoxide [51]. Peroxynitrite damages DNA and stimulates the DNA repairing enzyme poly(ADP-ribose) polymerase (PARP) to be released. However, it is important to mention that this enzyme requires significant energy for its activation, and therefore impairment of myocardial energetics is seen in patients with TTS [52]. Therefore, further studies are needed to investigate the role of a catecholamine surge in TTS development.

Several studies have shown that activation of β-adrenoreceptors stimulates the proliferation of neonatal cardiomyocytes [53], mesenchymal stem cells [54], as well as the differentiation of cardiac embryonic stem cells (ESC). These studies suggested that the stimulation of β-adrenoreceptors can promote the differentiation and proliferation of induced pluripotent stem cell-derived cardiomyocytes (iPSC-CMs). Li et al. showed that activation of adrenoreceptors enhances the differentiation of cardiomyocytes from iPSC-CMsh, which is regulated through the alpha1-receptors pathway [55]. Further studies are needed to confirm these results.

Another hypothesis of TTS pathogenesis is endothelial dysfunction, which may represent an essential link between stress and myocardial dysfunction. Endothelial dysfunction has been suggested as a plausible causative factor explaining the subepicardial and/or microvascular vasospasm. In a prospective observational study, authors investigated changes in endothelial function and concluded that 22 TTS patients demonstrated impaired flow-mediated vasodilatation compared to 21 matched controls (p = 0,016). Transient myocardial ischemia related to endothelial dysfunction and subsequent cardiomyocyte stunning might be the possible causes of the reversible LV dysfunction in TTS [56], [57]. Recently, plaque rupture and thrombosis followed by transient ischaemia were considered as one of the possible mechanisms of TTS development. The basis for this hypothesis was the detection of atherosclerotic plaques in some patients using optical coherence tomography in patients with TTS. However, Haghi et al. rejected this hypothesis as they detected no ruptured plaque in any patient [58]. Similar results were obtained 6 years later by Eitel et al. in a single-centre study [59]. In the meantime, it should be noted that obstructive coronary lesions can be observed approximately in 1 out of 10 TTS patients pointing to the possible association of TTS with CAD [60], [61].

TTS was also associated with a chronic inflammatory state [62] in a multicenter study, and increased endothelial and vasomotor dysfunction was found among TTS patients [57], [63]. To date, elevated levels of IL-6 and IL-10 in TTS have been explained by the fact that catecholamines activate endothelial cells and vascular smooth muscle cells, which produce these cytokines. In a rat model of hypoxic myocardial stress, Yamauchi-Takihara et al. found that several factors, including epinephrine, can induce IL-6 mRNA expression [64]. The correlation of IL-10 with elevated epinephrine levels was also shown in a study by T. Ohtsuka et al. [65]. Therefore, the inflammatory cascade may be proportionally enhanced by catechol-aminergic myocardial endothelial damage due to severe peripheral vasoconstriction.

The growth of TTS during the COVID-19 pandemic can not be overlooked and is explained by three leading theories to date:

1. The cytokine storm leads to increased release of tumour necrosis factor-α, IL-6, IL-1β, and catecholamines into the bloodstream, contributing to TTS development [66]. The exact mechanism of the effect of proinflammatory interleukins on catecholamine release is unknown. However, a systematic review has shown that a cytokine storm (observed in Covid-19) can lead to direct catecholamine toxicity and myocardial damage, contributing to TTS development [67]. This hypothesis is also supported by a clinical case of a patient with TTS who recovered after IL-6 inhibitor administration [68].
2. Activation of the sympathetic nervous system causes catecholamine-induced myocardial stunning and, as a result, TTS [69] and microvascular dysfunction [70].
3. An immense emotional stress caused by a cataclysm and a high mortality rate may trigger TTS development. In comparison, according to the US nationwide study, the highest incidence of TTS cases was identified in Vermont and Missouri (the Tropical Storm Irene and Joplin Tornado struck these states in the year of the study, and these natural disasters claimed hundreds of lives) [71].

Furthermore, the series of ongoing research on the pathogenetic mechanisms of TTS attempts to explain the development of the syndrome at the molecular level. In particular, overexpression of microRNA-16 and microRNA-26a specifically reduced baseline contractility of apical but not basal cardiomyocytes in vitro and functionally interacted to predispose to the cardiac depression seen in TTS [72]. Thus, it is vital to learn more about pathogenetic mechanisms of the condition to develop new ways of its prevention and treatment.

## 6 Classification

Today TTS is commonly divided into two clinical subtypes: primary and secondary (Fig. 1a). Primary TTS is a leading cause of hospital admission, commonly triggered by psychosocial stress, although emotional stress may not be identifiable in some cases. Secondary TTS develops among patients already admitted to hospitals due to other various conditions, such as asthma attack, surgical intervention, or severe physical trauma and may also involve drug-induced TTS (i.e., catecholamine stimulation, antidepressants, antiarrhythmics, radiation, and chemotherapy) [49], [73]. In addition, there is a clinically used classification based on the wall motion abnormality (WMA) location (Fig. 1b), which subdivides TTS into apical, mid-ventricular, basal, focal, and biventricular TTS [74].

**Fig. 1a:**
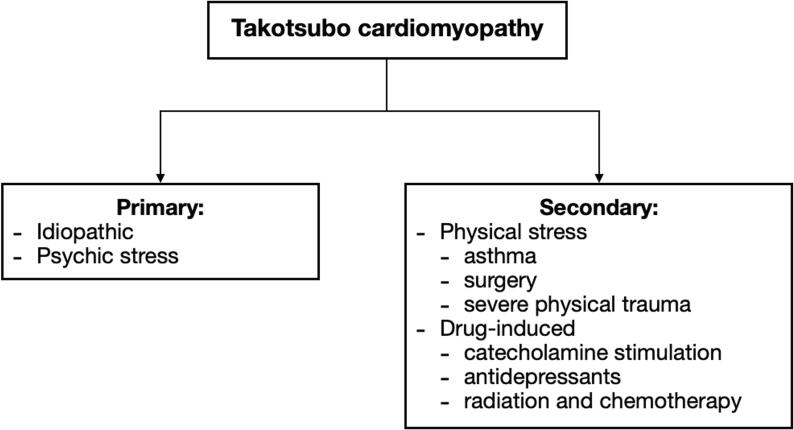
Classification of TTS based on etiology.

**Fig. 1b:**
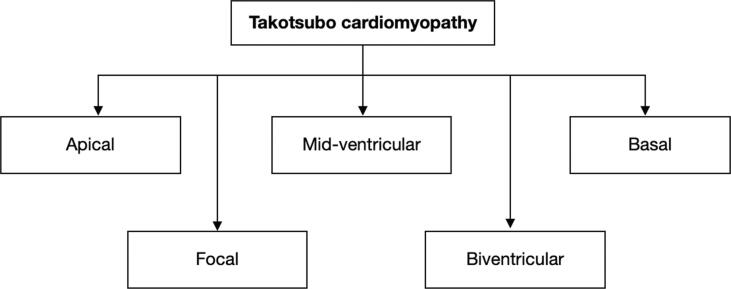
Classification of TTS based on the location of WMA.

## 7 Clinical profile: Typical vs atypical TTS patient

**Fig. 2:**
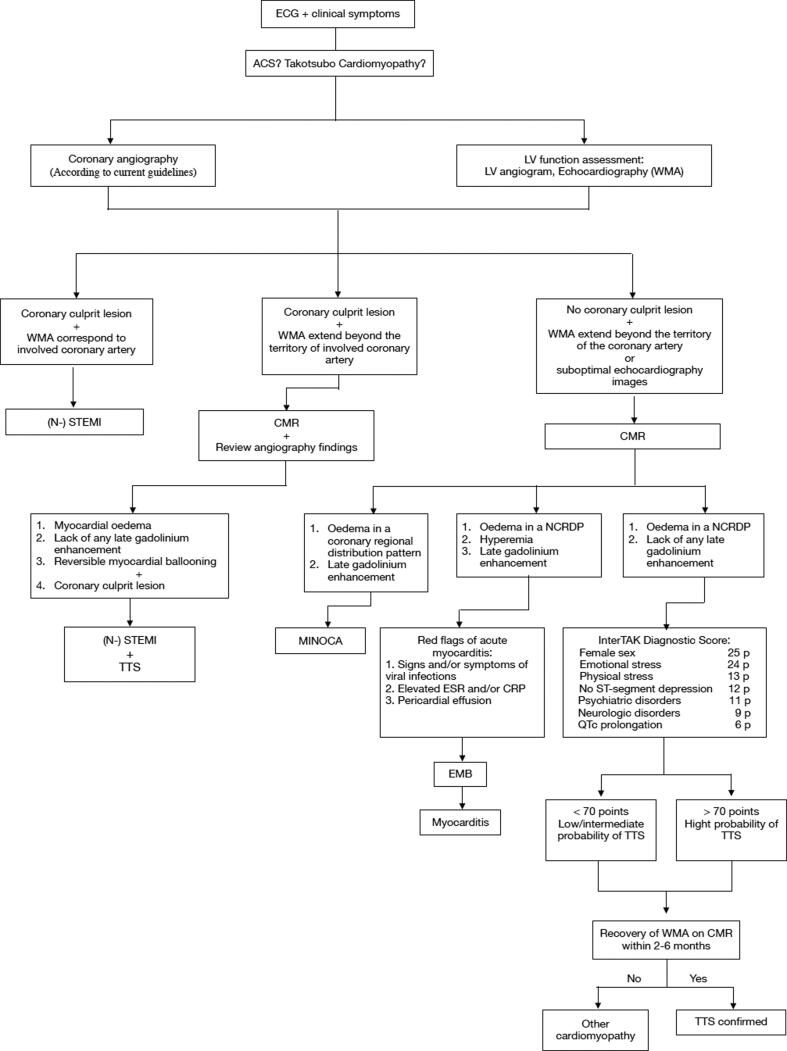
Diagnostic algorithm for undifferentiated chest pain and/or shortness of breath: Takotsubo Cardiomyopathy and/or Acute Coronary Syndrome. ECG – Electrocardiography, ACS – Acute coronary syndrome, LV – Left ventricle, WMA – Wall motion abnormalities, (N-) STEMI – (Non-) ST-elevation myocardial infarction, CMR – Cardiac magnetic resonance, NCRDP – Noncoronary regional distribution pattern, MINOCA – Myocardial infarction with nonobstructive coronary arteries, ESR – Erythrocyte sedimentation rate, CRP – C-reactive protein, EMB – Endomyocardial biopsy, TTS – Takotsubo cardiomyopathy, p – points.

As described above, a typical TTS patient is a postmenopausal woman over 50 years old with symptoms similar to myocardial infarction, such as sudden onset chest pain, dyspnea, or syncope, provoked by emotional stress and characterized by the development of apical ballooning. In the meantime, concomitant CAD is reported among patients with TTS, with a prevalence ranging from 10 to 29%. Therefore, patients with TTS and obstructive CAD are often misdiagnosed as a classical acute coronary syndrome, and differentiation can be challenging [75]. In addition, there are various scenarios where clinicians need to differentiate TTS from myocarditis, myocardial infarction with nonobstructive coronary arteries (MINOCA) (Fig. 2).

Underlying chronic conditions (e.g., diabetes mellitus, asthma), cerebrovascular events (e.g., ischemic stroke, transient ischemic attack), or seizures may often predispose to TTS, which underpins the role of extracardiac factors in the development of the disease. In this regard, symptoms may include atypical clinical manifestations (loss of consciousness, neurologic signs, or blood pressure dropping). Besides, TTS can manifest at a younger age, and patients are often admitted with the symptoms of acute heart failure and cardiogenic shock, pulmonary edema, or stroke [5].

According to the International Takotsubo Registry, among 1750 patients from 9 countries, atypical presentation, including younger age, neurologic symptoms, ST depression, and lower level of B-type natriuretic peptide (BNP), accounted for 20% of all cases, while outcomes and prognosis were comparable between patients with a typical and atypical presentation [76]. Therefore, notwithstanding a better understanding of TTS diagnosis, it is of critical value to closely monitor patients with both typical and atypical clinical presentation, as a diagnostic reasoning bias cannot be eliminated.

## 8 Diagnostics

TTS was first classified as primary cardiomyopathy in 2006 [77]. Since then, several diagnostic criteria have been proposed for TTS diagnosis. One of the most widespread diagnostic tools is the revised version of Mayo Clinic Diagnostic Criteria for TTS (2008), which incorporates transient wall-motion abnormalities, absence of a potential coronary culprit, myocarditis, and pheochromocytoma [63]. In addition, the European Society of Cardiology (ESC) combined up-to-date data and created the International Diagnostic Takotsubo Criteria (InterTAK diagnostic criteria) in 2018 [5], which included pheochromocytoma as a possible trigger and a secondary cause of TTS. Most importantly, coronary artery disease does not rule out TTS because of the possible coexistence of these two illnesses, and according to some data, ACS can instigate TTS [75], [78], [79], [80]. Therefore, in 2019 Heart Failure Association proposed the updated diagnostic criteria, where the presence of obstructive coronary lesion is no longer considered an exclusion, and among such patients, WMA may extend beyond the culprit lesion. Also, the ventricular systolic function recovery on imaging in 3–6 months was added to the criteria [81]. The comparison of the diagnostic criteria of Revised Mayo Clinic Diagnostic Criteria, InterTAK, and Heart Failure Association criteria 2019 are presented in Table 5.

**Table 5:** Comparison of the main diagnostic criteria.

Diagnostic criteria	Revised Mayo Clinic Diagnostic Criteria 2008 [63]	InterTAK Diagnostic Criteria 2018 [5]	Heart Failure Association criteria 2019 [81]
**Transient Wall Motion Abnormalities**	+	+	+
**Stress as a trigger**	+	+	+
**Neurological disorders as a trigger**	*not specified*	+	*not specified*
**New ECG abnormalities**	+	+	+
**Cardiac biomarkers elevation**	+	+	+
**Serum natriuretic peptide elevation**	*not specified*	+	+
**Coronary artery disease**	Absence of obstructive coronary disease	Significant coronary artery disease is not a contradiction	Absence of culprit atherosclerotic CAD to explain the pattern of temporary LV dysfunction observed
**Pheochromocytoma**	Absence	May serve as a trigger	*not specified*
**Absence of Infectious myocarditis**	+	+	+
**Postmenopausal women**	*not specified*	Predominantly affected	*not specified*
**Systolic function recovery**	*not specified*	*not specified*	Within 3–6 months

Table summarising three main diagnostic criteria: Revised Mayo Clinic Diagnostic Criteria 2008, InterTAK Diagnostic Criteria 2018, Heart Failure Association criteria 2019.

ECG — Electrocardiography; CAD – coronary artery disease; LV – left ventricle.

It should be noted that patients with TTS are presented with the same set of clinical features as patients with ACS. Therefore, it is assumed that the characteristic clinical signs and ECG patterns allow the diagnosis of ACS and coronary angiography per the current guidelines [82] to exclude myocardial infarction in the first place. Based on InterTAK diagnostic criteria, we propose a practical diagnostic algorithm, modified after Napp and Bauersachs [83], including invasive visualisation of coronary arteries, echocardiography, and InterTAK Diagnostic Score (Fig. 2). Subsequently, three clinical scenarios are possible based on the results of coronary angiography and echocardiography.

The first scenario is the presence of coronary culprit lesion plus WMA corresponding to the involved coronary artery, which confirms (N-)STEMI. The second scenario is characterised by the coronary culprit lesion with WMA, which extends beyond the involved coronary artery, which may be due to the coexistence of two conditions, both (N-)STEMI and TTS. Finally, the third scenario is the absence of coronary culprit lesion, but the presence of WMA, which extends beyond a single epicardial coronary distribution, which subsequently requires a differential diagnosis of MINOCA, myocarditis, and cardiomyopathy, including TTS, by performing cardiac magnetic resonance (CMR) [82].

The laboratory and various instrumental methods for the diagnosis of TTS have different diagnostic values.

### 8.1 Biomarkers

In addition to slightly elevated Troponin T, as a result of acute myocardial damage, Takotsubo cardiomyopathy is characterised by a marked increase in plasma levels of B-type natriuretic peptide (BNP) and N-terminal prohormone of B-type natriuretic peptide (NT-proBNP) with its subsequent peak elevation within 24–48 h after onset of initial symptoms. Furthermore, the ratio of peak levels of NT-proBNP/troponin T was demonstrated to be most accurate in identifying patients with TTS among those admitted with (N)-STEMI [84].

Besides, in an acute phase of TTS, a higher elevation of growth differential factor-15 and proinflammatory cytokines has been described compared to STEMI. On the other side, many cardiac markers, such as troponins, creatine kinase-MB, or markers of vascular stress (i.e., copeptin), are lower in TTS patients than in STEMI [85]. Biomarkers of chronic Takotsubo stage have also been reported. According to Scally et al., high sensitivity troponin I, IL-6, IL-8, and BNP may be identified five months after TTS onset [62]. The summary data of biomarkers in TTS at admission versus STEMI and in the chronic phase of TTS are presented in Table 6.

**Table 6:** Main biomarkers in the acute and chronic phase of TTS [85].

Biomarkers	Acute phase (at admission)	Chronic phase (at 5 month)
**Cardiac injury**	NT-proBNP/troponin T ratio, BNP, Troponins, hs-TnT/CK-MB	Troponin I; BNP
**Immune response/inflammation**	Il-2; Il-4; Il-6; Il-10; TNF-a; Leukocytosis and high CRP;	Il-6; Il-8
**Growth factor**	Growth differential factor-15; endothelial growth factor	*Not described*
**Markers of vascular stress**	Copeptin	*Not described*
**Messenger markers**	microRNA-16 and microRNA-26a specifically reduced baseline contractility of apical cardiomyocytes	*Not described*

Table summarising main biomarkers in both acute and chronic phases of TTS.

NT-proBNP – N-terminal prohormone of B-type natriuretic peptide; BNP – B-type natriuretic peptide; hs-TnT – Troponin T, high sensitivity; CK-MB – Creatine kinase-MB; TNF-a – Tumour necrosis factor α.

### 8.2 Electrocardiography (ECG)

ECG has a low value for TTS diagnostics due to its similar criteria to acute coronary syndrome. In TTS, ECG patterns of ST-elevation (STE) or T-wave depression (if present) demonstrate dynamic changes with the gradual resolution of ST-segment elevation, subsequent T-wave inversion, and QTc prolongation over days to weeks [86]. All these findings primarily require the exclusion of myocardial infarction. In 2016 a retrospective analysis was performed that included 200 patients with TTS and 200 patients with ACS. The authors proposed a practical ECG algorithm that diagnoses STE-TTS, STEMI, NSTE-TTS, or NSTEMI with >95% specificity, stating that ECG criteria could distinguish the two entities [86]. However, coronary angiography and ventriculography are needed to confirm the diagnosis.

Also, some differences in ECG features among AA patients with TTS have been described with the prevalence of diffuse T-wave inversion and more prolonged QTc [87].

### 8.3 Echocardiography

In contrast, echocardiography is essential in visualising WMA extending beyond the territory of the involved coronary artery, which is the main indication of TTS. Therefore, echocardiography criteria may include the following [76], [88], [89], [90]:

a) LV regional WMA, which frequently involves apical and midventricular segments with the ballooning pattern. Conversely, the basal myocardial segments are often hyperkinetic. As a rule, WMA extends beyond a single epicardial coronary distribution;
b) Transient LV dysfunction, often with a circumferential pattern, and a complete resolution of LV systolic function over 3–6 months;
c) Improvement in E/e ratio during follow-up may be considered an additional useful indicator of LV function recovery;
d) Distal Left Anterior Descending artery flow visualisation.

Moreover, echocardiography is a valuable method for diagnosing and visualising acute and long-term TTS complications, such as wall rupture, acute mitral regurgitation, left ventricle outflow tract obstruction (LVOTO), and RV involvement, pericardial effusion, thrombus formation, heart failure [91], [92].

### 8.4 Coronary angiography and left ventriculography

Although coronary anatomy in most TTS cases is normal or near-normal, it is crucial to evaluate coronary arteries in TTS diagnostic workup to exclude alternative diagnoses [93]. It should be noted that a one-vessel obstructive coronary artery disease does not exclude TTS, as LV wall motion abnormalities are often beyond the vascularisation zone of the involved vessel in TTS (Fig. 2). Conversely, nonobstructive lesions still may cause ACS [91], [94].

When echocardiography is inconclusive or unavailable, the angiographic biplane left ventriculography helps evaluate both LV function and its morphology by identifying the characteristic TTS patterns [90]. According to Desmet W et al., in almost one-third of observed TTS cases, the presence of a “very small zone of contractility in the most apical portion of the LV” coined as the “apical nipple” was identified during the biplane left ventriculography. At the same time, this sign was not seen among STEMI cases [95]. In addition, ventriculography helps identify the fluoroscopic “hawk’s beak” sign, which represents mid-ventricular region systolic ballooning with preserved LV apical contractility in TTS and can be used as an early diagnostic marker in differential diagnosis with ACS [96], [97].

The biplane left ventriculography is recommended along with cardiac catheterization through LV opacification in case of suspected ACS and non-obstructive coronary lesions [89]. Besides, left ventriculography may be helpful in detecting complications including acute mitral regurgitation or LV apical thrombi, as well as in determining intraventricular pressure gradients to detect LVOTO, particularly in haemodynamically unstable patients [98], [99].

### 8.5 Cardiac magnetic resonance (CMR)

CMR is an essential instrumental method due to its value for differential diagnosis and exclusion of myocarditis or myocardial infarction. The main CMR signs of TTS are the absence of late gadolinium enhancement and the presence of edema in a noncoronary regional distribution pattern (NCRDP). Complete CMR criteria include the following [100], [101], [102], [103]:

a) LV dysfunction in a noncoronary regional distribution pattern with or without RV involvement;
b) Myocardial edema located in the segments with WMA (akinesia or hypokinesia);
c) Absence of significant necrosis or fibrosis;
d) Absence of high-signal areas (>5 SD above normal) in late gadolinium enhancement images;
e) Complete normalisation of LV ejection fraction on the follow-up CMR imaging.

Therefore, CMR is the gold standard for determining myocardial status [104]. In the future, the broader use of CMR may help in the early diagnosis of TTS and prediction of complications at admission.

### 8.6 Coronary computed tomography angiography (CCTA)

CCTA provides information on both coronary arteries and regional LV contraction [105]. Therefore, it may be recommended as an alternative diagnostic method in patients with suspected TTS and co-existing life-threatening conditions such as terminal malignancy, intracranial bleeding, sepsis since coronary angiography can add a considerable risk for complications [106]. In addition, considering quick accessibility to CCTA as a less invasive modality, it can be promptly used in the emergency settings for stable patients with low suspicion for ACS and strong clinical and echocardiographic findings compatible with TTS [107].

Beyond coronary artery disease itself, multislice computed tomography (MSCT) helps detect myocardial fibrosis and late iodine enhancement, thus is effectively used as an additional diagnostic tool in differential diagnosis with myocardial infarction and acute myocarditis [108], [109]. The method through multiplanar imaging also provides greater visualisation of LV thrombi, a not so infrequenlty seen TTS complication [110].

Routine CCTA can also provide a quantitative measurement of the coronary artery inflammation process by calculating the weighted pericoronary fat attenuation index (pFAI) [111], [112]. Galbazzi et al. have demonstrated an association between the significantly higher values of pFAI and coronary artery inflammation among patients with MINOCA and TTS than in the controls undergoing CCTA for atypical chest pain and no signs of obstructive coronary artery disease and no follow-up cardiac events [113]. Earlier, in a post-hoc analysis by Oikinomou et al., high values of pFAI were associated with increased all-cause and cardiac mortality and, therefore, can enhance prediction of cardiac risks along with coronary calcium in the current CCTA assessment [114]. Integration of pFAI into standard CCTA may advance cardiac risk-stratification in the presence of nonobstructive coronary plaques.

### 8.7 Cardiac nuclear imaging

Cardiac nuclear imaging has been successfully used in evaluation of myocardial perfusion and its metabolic activity [115]. It involves single-photon emission computed tomography (SPECT), positron emission tomography (PET), scintigraphy, combined PET/CMR, and combined PET/computed tomography (CT) scanners [116], [117].

SPECT demonstrates perfusion reduction due to regional myocardial wall thinning at the apex, which helps diagnose TTS [118]. Furthemore, the adrenergic hyperactivity seen among TTS patinets leads to “decreased uptake and increased washout of 123I metaiodobenzyl guanidine (MIBG) from the heart” during 123I-MIBG scintigraphy [32], [119]. Therefore, simultaneous use of 123I-MIBG and myocardial perfusion scintigraphy can help in the differential diagnosis of TTS from ACS in case regional innervation and myocardial scarring are parallel and matched [91].

Cardiac positron emission tomography (PET) features a mismatch between impaired myocardial glucose metabolism and normal myocardial perfusion in TTS, also known as «inverse flow metabolism mismatch» and can be employed in differential diagnosis with ACS [120]. The abnormal glucose metabolism and impaired sympathetic innervation detected by PET and 123I-MIBG scintigraphy, respectively, might help diagnose TTS in patients with delayed presentation during the LV wall motion recovery [121], [122].

Combined PET/CMR scanners can also provide a more comprehensive and non-invasive modality in the differential diagnosis of TTS from myocarditis, which is critical as the clinical course and prognosis vary considerably [123], [124], [125]. Combined PET-CT/CMR imaging is more informative than CMR alone in differential diagnosis with myocarditis, as it helps in quantifying the extent of inflammation along with the late gadolinium enhancement seen in patients with suspected myocarditis [126].

### 8.8 Autopsy

Some microscopic changes of TTS have been found in recent studies [127], [128].

a) An extensive area of myocardial thinning;
b) Evidence of Lewy body-like cytoplasmic inclusions in dorsal nuclei of the nervus vagus;
c) Interstitial oedema with the accumulation of mononuclear leukocytes, lymphocytes, macrophages, polymorphonuclear leukocytes, mast cells, and eosinophils;
d) Focal myocardial fibre degeneration and necrosis with foci of inflammatory cells (histiocytes, plasma cells, and polymorphonuclear leukocytes);
e) Contraction bands with or without overt myocyte necrosis.

Researchers hypothesise that it is possible to make a differential diagnosis at autopsy and to diagnose TTS. Therefore, it is crucial for forensic medical examinations, treating physicians, and close relatives to understand the actual cause of the patient's death. Unfortunately, to date, there are few reports on the pathomorphological features of the heart (both macroscopic and microscopic), which means that more research is needed to look more closely at the pathophysiology of TTS and to identify independent predictors of fatal complications.

## 9 Management

### 9.1 TTS without any complications

There are currently several approaches to TTS pharmacotherapy. Table 7 summarises the results of significant clinical trials, including authors, years of publication, number of patients participating in the study, groups of prescribed drugs, follow-up time, side effects, and summary. The most commonly used therapy is a combination of angiotensin-converting enzyme (ACE) inhibitors and beta blockers. Despite the rapid recovery of left ventricular function with the beta-blockers monotherapy [129], the latter did not decrease mortality [130]. Nevertheless, data shows that the combination of beta blockers and ACE inhibitors can be more appropriate and beneficial [131], as ACE inhibitors prevent vasospasm, consequently reducing hypertension, and beta blockers inhibit the Gs-protein metabolic pathway preventing recurrence of TTS development.

**Table 7:** Drug therapy for TTS.

Medication group	Adverse Reactions	Conclusion	Number of patients	Follow-up duration	Authors, year of publication
**Beta-blockers and ACE inhibitors** **(17.1% of patients had a history of using antidepressants)**	3% — ventricular tachycardia 1,3% — ventricular thrombus 0,2% — rupture of the ventricular	ACE inhibitors improve annual survival over a year, and Beta-blockers did not show any significant effect	1750 (retrospective study)	Ten years	Templin et al. 2015 [75]
**Aspirin, clopidogrel, statins, beta-blockers, ACE inhibitors**	2,8% — death 2,8% — congestive heart failure	Refuse antiplatelet therapy; there are repeated hospitalizations (2,8%)	117	One year	Yayehd et al. 2016 [133]
**Antiplatelet therapy, beta-blockers, ACE inhibitors, or statins**	7,3% — death 7,3% — apoplexy 26,7% — congestive heart failure	Antiplatelet therapy is effective. No significant effect of beta-blockers and statins is shown	206 (retrospective study)	Before patient’s discharge	Dias et al. 2016 [132]
**Beta-blockers, ACE inhibitors**	28% — relapse after six years 11% -- dyspnoea 14% — chest pain	Reduction of TTS relapse rate	1664 (multiple *meta*-regression analysis)	Two years	Brunetti et al. 2016 [131]
**Beta-blockers**	2% — death (early therapy) 2,4% — death (therapy during hospitalisation)	Beta-blockers show equal efficacy both in the early stages and in later stages of the disease	2110 (retrospective study)	From the start of medication to condition improvement/death	Isogai et al. 2016 [130]
**Levosimendan**	No serious complications observed	Levosimendan infusion at 0.1 mcg/kg/min with its loading dose of 10 mcg/ kg added to standard medication therapy shortened the recovery time of the myocardium.	42 (retrospective study)	Hospitalisation period	Yaman et al. 2016 [231]
**Enoxaparin, Warfarin**	2,2% — left ventricle thrombosis	Effective in the acute phase. It could be used up to 3 months for apoplexy prevention.	12	984 days (about three years)	Santoro et al. 2017 [147]
**Catecholamines**	3,2% — death	The use of catecholamines for maintaining blood circulation increases the risk of death	114	Four years	Ansari et al. 2018 [232]
**Beta-blockers**	26,6% — death 7,7% — death resulting from heart diseases	Hemodynamical and ECG monitoring of the condition of patients is necessary. Preference should be given to short-acting beta-blockers	154 (retrospective study)	One year	Madias et al. 2018 [233]
**Beta-blockers**	95% — improvement of LV systolic function	Complex treatment of TTS with beta-blockers restores the systolic function of the left ventricle in a significantly higher number of patients	124 (retrospective study)	Three years	Ghalyoun et al. 2019 [129]
**Beta-blockers, ACE inhibitors**	7,8% — arrhythmia 11,9% — cardiogenic shock 14,6% — thromboembolism 4% — transfer to the intensive care unit	Combination of beta-blockers and ACE inhibitors is not an effective and safe method of managing TTS	103	Five years	Kummer et al. 2020 [234]

Table summarising studies on pharmacological drugs used for TTS treatment.

ACE inhibitors — Angiotensin-converting enzyme inhibitor, TTS — Takotsubo cardiomyopathy, ECG — Electrocardiography, LV — Left ventricle.

Also, some studies suggest that haemodynamically stable patients should be given anticoagulants (heparin) or dual antiplatelet therapy (aspirin and clopidogrel) to prevent complications and recurrent TTS [132], [133]. However, there is controversy about the timing of their use, with some authors emphasising their use in the presence of a thrombus and others saying that they should be given as soon as the diagnosis of TTS has been made. Thus, more research is needed to determine the role of anticoagulants and dual antiplatelet therapy in TTS treatment.

It has been shown that single antiplatelet therapy with aspirin or dual therapy with aspirin plus clopidogrel can reduce major adverse cardiovascular events during hospital admission, including stroke [134]. However, in a study by Bertaina et al., it was shown that aspirin did not reduce the risk of recurrent TTS among patients discharged without subsequent use of beta-blockers and ACE inhibitors [135]. The same results were obtained by researchers who published the first *meta*-analysis to investigate the efficacy of drug therapy after patients were discharged [136]. A second *meta*-analysis and systematic review protocol studying the effect of aspirin on Takotsubo Syndrome have now been published [137]. In 2020, a study involving 1,533 patients with TTS demonstrated that aspirin use had no significant effect on the prognosis and development of complications after discharge [135].

### 9.2 TTS and heart failure (HF)

HF is treated with standard therapy, including beta blockers in a compensated condition, ACE inhibitors, or angiotensin receptor blockers (ARBs), and diuretics [138]. Respiratory support may be used if necessary. According to Kumar S et al., beta blockers reduce the incidence of cardiac rupture in TTS [139]. However, they are contraindicated in decompensated HF. The duration of treatment, on average, is up to 4 weeks if there is a visible improvement of the heart contractility, but treatment duration may vary depending on comorbidities and other risk factors. A study by Abanador-Kamper et al. concluded that treatment of HF is most effective during the first 1–2 months after hospital admission [140].

### 9.3 TTS and cardiogenic shock (CS).

According to 2020 Mayo Clinic data, 11% of patients develop CS in the first 72 h after admission, and 20–25% develop LVOTO [141].

1. *Without left ventricular outflow tract obstruction (LVOTO).*
These patients usually develop hypotension. Either sympathomimetics or inotropic therapy can restore blood pressure. Given the pathogenesis of TTS, sympathomimetics (for example, norepinephrine or ephedrine) can worsen the patient's condition, so dobutamine/dopamine is the drug of choice because of their positive inotropic effect. If the patient develops moderate to severe LVOTO, inotropic therapy should be discontinued. If there is evidence of decreased blood supply to internal organs and persistent hypotension, vasopressor therapy can be used, which increases cardiac output and thus blood pressure. Severe hypotension and cardiogenic shock can lead to left ventricular dysfunction. In this case, mechanical circulatory support must be used to keep patients alive [142].
2. *With left ventricular outflow tract obstruction (LVOTO).*
As already mentioned, inotropic therapy must be discontinued. Fluid resuscitation and beta-blockers are the therapy of choice in this case (in the absence of pulmonary congestion). In addition, an alpha-agonist (phenylephrine) can be used to increase the blood pressure and therefore the afterload, which may improve the patient's condition. Mechanical circulatory support (particularly intra-aortic balloon pump) should be used if the conservative therapy is ineffective [143].

Levosimendan, a non-catecholamine inotrope that does not increase myocyte uptake of cytoplasmic oxygen, is a rational therapeutic option in cardiogenic shock associated with TTS. In addition to its direct action as a calcium-sensitising agent, levosimendan has been found to mediate the opening of adenosine triphosphate (ATP)-dependent potassium channels in vascular smooth muscle cells [144]. Through this mechanism, levosimendan causes increased blood perfusion in crucial organs and systemic vasodilation. Furthermore, levosimendan also opens ATP-dependent potassium channels on the inner mitochondrial membrane, which is considered a cardioprotective effect of the drug [145].

**Fig. 3:**
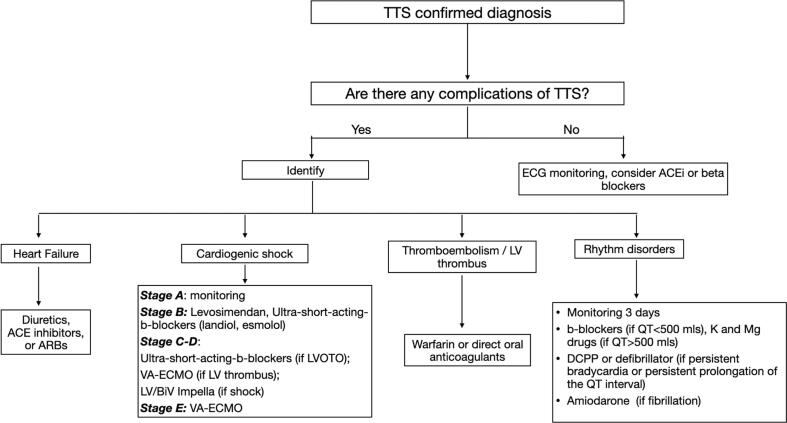
Treatment of TTS complications*.* TTS - Takotsubo cardiomyopathy, ECG - Electrocardiography, ACEi - Angiotensin-converting enzyme inhibitor, ARB - Angiotensin II receptor blocker, LVOTO - Left ventricular outflow tract obstruction, VA-ECMO -Venoarterial extracorporeal membrane oxygenation, LV - Left ventricle, BiV - Biventricular, DCPP - Dual-chamber permanent pacemaker.

Depending on the stage of CS [146], relevant treatment measures should be applied (Fig. 3) [83]:

1. Stage A (At risk): no tachycardia, hypotension, and normal lactate values. At this stage, only close monitoring of the patient's condition is necessary;
2. Stage B (Beginning): hypotension (relative), tachycardia without hypoperfusion, and normal lactate values. Levosimendan and ultra-short-acting beta blockers (landiolol, esmolol) should be administered;
3. Stage C (Classic): hypoperfusion, elevated lactate values, cardiac index < 2.2 L/min/m*2. In the absence of LVOTO, venoarterial extracorporeal membrane oxygenation (VA-ECMO) is required if there is a left ventricular thrombus, and LV or BiV Impella if no thrombus is found. If LVOTO is developed, the inotropic therapy should be discontinued, and ultra-short-acting beta blockers (landiolol, esmolol) should be prescribed instead. However, if the patient does not improve, VA-ECMO or LV/Biventricular (BiV) Impella should be started, depending on the presence or absence of thrombus, respectively;
4. Stage D (Deteriorating): hypoperfusion and deterioration (Not refractory shock). Treatment is the same as for stage C;
5. Stage E (Extremis): hypoperfusion, deterioration, refractory shock. VA-ECMO is needed at this stage;

### 9.4 TTS and thromboembolism

The most commonly used drug for treating thrombosis in TTS is warfarin, a vitamin K antagonist. The optimal duration of treatment is three months, but depending on the size and location of LV thrombi, it can be prolonged [147]. To date, no studies are investigating the role of novel oral anticoagulants, so the question of their efficacy remains unresolved.

### 9.5 TTS and rhythm disorders

According to 2018 data, the prevalence of arrhythmias among 286 patients with TTS demonstrated that ventricular tachycardia (VT) and ventricular fibrillation (VF) accounted for two-thirds of the arrhythmic episodes [148]. In general, arrhythmias in TTS last for several days and stop under the influence of beta-blockers. However, there are a few recommendations that practising physicians should follow when managing a patient with TTS-associated arrhythmia [149]:

1. Telemetry monitoring should be carried out for at least three days (1–2 days if the QT interval is restored);
2. Beta-blockers should be discontinued, and potassium and magnesium should be given in patients with a QT interval > 500 ms;
3. It is advisable to be alert to the list of antiarrhythmic drugs that prolong QT interval. Although intravenous amiodarone is the most commonly prescribed medication for treating life-threatening arrhythmia, a few cases of torsade de pointes have been reported [150], [151]. Early detection of concurrent electrolyte disturbances, careful QT interval monitoring, and awareness of drug interaction profiles can be helpful;
4. In the case of torsades de pointes, intravenous magnesium is the first-line pharmacological treatment approach. In patients with prolonged QT interval and unresponsive to magnesium treatment, isoproterenol was shown to prevent torsades de pointes. Synchronised cardioversion is the preferred method for hemodynamically unstable patients who have a pulse. Patients with pulseless torsades should be defibrillated [152];
5. A dual-chamber permanent pacemaker or defibrillator may be considered in persistent bradycardia or persistent prolongation of the QT interval. Bradyarrhythmias can be dangerous if they lead to complete atrioventricular heart block (AV-block). In the Stiermaier T et al. study, researchers suggested that patients in this situation need a permanent ventricular pacemaker with long-term follow-up, indicating that bradyarrhythmias in TTS require implantation of a permanent pacemaker [153]. In contrast, according to this study, polymorphic ventricular arrhythmias were restricted to the acute and subacute phase until repolarisation recovery, suggesting a temporary treatment approach (e.g., transient pacemaker, wearable defibrillators). However, further studies on device implantation in TTS are needed to confirm these results.

## 10 What lies ahead for the TTS treatment?

NACRAM trial is a multicenter, randomised, placebo-controlled trial consecutively testing the early use of intravenous N-acetylcysteine followed by oral administration of ramipril for 12 weeks in TTS management. The use of these medications is reasoned by their inhibiting effects on nitrosative stress and expression of the activator thioredoxin interacting protein. Both processes are presumably related to TTS pathogenesis. NACRAM evaluates the reduction of myocardial edema on magnetic resonance imaging of the heart. It also analyses the improvement of LV systolic function measured by global longitudinal strain on echocardiography, evaluates the quality of life and inflammation markers. NACRAM is likely to become the first prospective study that will evaluate treatment options of acute attacks of stress-induced cardiomyopathy [154].

Also, hormone therapy is currently being researched. The advantages of estradiol are still being studied and assessed in treating the chronic phase of TTS. Animal experimental trials have demonstrated a significant reduction in stress-induced LV dysfunction and arrhythmias. It indicates the importance of the sex hormone in the clinical management of TTS after complete recovery of LV function established by echocardiography. It has also been demonstrated that oestrogen has a cardioprotective effect against myocardial damage in the context of ischemia or reperfusion [155]. However, the available data about the role of estrogens is limited and contradictory. For instance, Möller et al. demonstrated no difference in sex hormones concentration among postmenopausal females with TTS and ACS, nor did they identify a protective effect of estradiol among those females under hormone replacement therapy (HRT) [156]. Further studies are needed to evaluate the role of hormones and the potential benefits of HRT in TTS.

To date, mineralocorticoid receptor antagonists and angiotensin-neprilysin receptor inhibitors (sacubitril/valsartan), which are effectively used in patients with chronic systolic heart failure, have not been studied among patients with TTS. However, these drugs may be of scientific interest for further research [157], [158].

A randomised interventional controlled trial involving 90 patients who have recovered from TTS is currently underway. The researchers are conducting a three-arm pilot trial of early rehabilitation, including standardised physical exercise training, cognitive behavioural therapy, and comparing the results with the standard clinical care. Key indicators are the “restored cardiac energetic status assessed by 31P-magnetic resonance spectroscopy, cortisol awakening response, global longitudinal strain by echocardiography, and the 6-minute walk test” [159].

## 11 Complications and their possible predictors

The most common complications of TTS are cardiogenic shock, rhythm disturbances, and thrombosis. We established the most likely independent predictors of various complications based on the data from multicenter studies, *meta*-analyses, and clinical cases (Table 8).

**Table 8:** Independent predictors for TTS complications.

Complication	HR (bpm)	BP (mmHg)	ST-changes	T changes	QT-interval (ms)	Peak NTproBNP or BNP (pg/mL)	Peak troponin (ng/mL)	AB or dyskinesia, akinesia, hypokinesia	Basal function	EF (%)	Reference
**VF**	100	94/70	—	Elevation (V2-4)	728	3.192 (NT proBNP)	—	Dyskinesia	Hyper	—	Taguchi M et al. 2020 [235]
**VF**	110	147/98	Elevation (II, III, aVF, V3-V6)	—	—	3431.5 (BNP)	0.813	Dyskinesia	Hyper	—	Mizutani K et al. 2020 [236]
**VF**	—	104/73	Normal	inversion (V4-V6)	539	—	0.143	Akinesia	Hyper	53	Wakatsuki D et al. 2020 [237]
**VF**	153	170/100	Depression (V4-V6)	inversion (V3-V5)	705	145. (BNP)	—	Hypokinesia	Hyper	—	Ishida T et al. 2017 [238]
**AF**	—	110/65	Depression	inversion (V2-V5)	520	1437 (NT proBNP)	670	Akinesia	Hyper	30	O’Brien et al. 2021 [239]
**AT and ischaemic stroke**	73	110/70	Elevation (V1-V6)	—	496	—	937	Akinesia	Hyper	38	Iuliano G et al. 2021 [240]
**AF**	114	133/64	—	inversion (V5-V6)	—	—	0.423	AB and akinesia	—	30	Sattar Y et al. 2020 [241]
**LVOTO**	110	90/60	Elevation (III – aVF)	—	—	3254 (BNP)	5	Akinesia	—	30	Attisano T et al. 2020 [242]
**AT**	110	100/60	Elevation (I-III, aVL, aVF, V2-V6)	—	—	—	2,2	Hypokinesia	—	40	Herath H et al. 2017 [243]
**AT**	—	—	Normal	inversion (all leads)	More than normal	—	3827	Akinesia	Hyper	36	Pongbangli N et al. 2019 [244]
**AT**	118	119/75	Elevation (II, III, aVF, V3-V6)	—	Normal	5435 (NT proBNP)	More than normal	Akinesia	Hyper	—	Nonaka D et al. 2019 [245]
**LVT**	—	—	Elevation (V3-V5)	inversion	Normal	—	528	Akinesia	Normal	—	Y-Hassan S et al. 2019 [246]
**Hepatic artery thrombosis**	120	—	Elevation (V2)	—	—	1660 (BNP)	0,45	Hypokinesia	—	15–20	Luu L et al. 2020 [247]
**LVT**	165	60/30	Elevation (II-III, aVF, V3-V6)	—	—	—	500	Akinesia	—	20	Joki T et al. 2020 [248]

Table summarising studies on the independent predictors for various TTS complications.

Hyper — Hypercontraction, VF — Ventricular fibrillation, AF — Atrial fibrillation, AT — Apical thrombus, LVT — Left ventricular thrombus, HR — Heart rate, BP — Blood pressure, EF — Ejection fraction, AB — Apical ballooning, LVOTO — Left ventricular outflow tract obstruction.

Independent predictors of cardiogenic shock [160] :

a. Male;
b. Asian or Hispanic;
c. Comorbidities, including congestive heart failure, chronic lung condition, and chronic diabetes.

Independent predictors of LV thrombus [94] and thromboembolic complications [161] :

a. Apical ballooning TTS;
b. Previous vascular disease;
c. LVEF ≤ 30%;
d. First WBC > 10 × 10*3 cells/μL;
e. Troponin I level > 10 ng/mL.

Independent predictors of cardiac arrest [162] :

a. High peak brain natriuretic peptide (BNP) values;
b. ST-segment elevation;
c. Atrial fibrillation;
d. High C-reactive protein.

Independent predictors of atrial fibrillation [163] :

a. Peak BNP 1620 ± 1459 ng/L;
b. Peak CRP 116 ± 96 mg/L;
c. LVEF 48 ± 15 %.

Independent predictors of ventricular fibrillation (VF):

a. Increased heart rate and blood pressure;
b. T-wave inversion (V4-V5);
c. QT interval prolongation;
d. Left ventricular basal hypercontraction.

A recent study showed that neutrophil/lymphocyte ratio is an independent predictor of hospital-acquired complications, including pulmonary edema, cardiogenic shock, death, stroke, and LV thrombi [164]. However, further studies are needed to determine the diagnostic and prognostic role of the general blood test.

Interleukins (IL) are hypothesised to influence TTS development, and their role as independent predictors of complications is also considered. For example, in 2016, Santoro et al. found that elevated levels of IL-6 and IL-10 on admission to hospital correlated with a high incidence of in-hospital complications. Patients with IL-6 > 51 pg/ml and/or IL-10 > 1.3 pg/ml were more likely to develop cardiogenic shock, pulmonary oedema, stroke, and recurrent TTS [165]. It has also been reported that IL infusion may contribute to the development of TTS [166]. Based on a study that involved 136 patients, the levels of anti-inflammatory cytokines (IL-2, IL-4, IL-10), two inflammatory cytokines (tumour necrosis factor (TNF)-a and interferon gamma (INF-g)) and the chemo-attract component epidermal growth factor (EGF) were higher among patients with TTS [167]. The higher levels of anti-inflammatory interleukins (IL-2, IL-4, and IL-10) found in TTS can be explained by the presence of macrophages M2 surrounding the damaged tissue [168]. For example, Nef et al. in their study of myocardial biopsy samples from TTS, immunohistochemical staining revealed the presence of several extracellular macrophage clusters (CD68) both in the acute phase and in the “recovered” myocardium [169]. As known, macrophages are cells of the immune system that can induce and suppress inflammation by secreting different groups of molecules.

In comparison to ACS, lower levels of IL-6 and higher levels of TNF-a and INF-g were found in patients with TTS, indicating a different response of cellular molecules in the two pathological processes. Researchers suggested that the higher levels of TNF-a and INF-g in patients with TTS may be related to their ability to remove damaged cells, while the higher levels of IL-6 in patients with ACS may indicate the process of atherosclerosis [167].

## 12 Prognosis

TTS is usually favourable and resolves with complete recovery in 4–8 weeks in>90% of patients. Relapses are rare. Thus, in a study by Templin et al. relapse rate was 1.8% per year [75]. A *meta*-analysis of 31 studies involving 1664 patients reported a 5% relapse after six years [170]. In a *meta*-analysis involving 3513 patients, Kato et al. reported a relapse rate from 0 to 10% [171]. Furthermore, in a study by Lau et al. with 519 patients, TTS recurred in 7.5% of patients over 5.2 years of follow-up [172].

Risk factors for developing relapses are severe LV dysfunction, increased susceptibility to emotional stress, dynamic fluctuations in the sensitivity of cardiac adrenergic receptors, and women over 50 years old [173]. Moreover, long-term mortality in TTS patients was significantly higher compared with the matched STEMI patients (24.7% vs. 15.1%, 95% confidence interval 1.07–2.33; P = 0.02). Masculinity, high Killip grade on admission, and diabetes mellitus were identified as independent predictors of mortality in TTS patients [174]. As such, preventive measures should be required to reduce the risk of complications and TTS recurrence.

According to Matabuena Gomez-Limon J et al. (1.8–6.6 years), long-term consequences include LV wall movement abnormalities, myocardial infarction, recurrent events of TTS, heart failure, and others [175]. Furthermore, independent predictors of in-hospital complications, according to Bento D et al. (234 patients), include chronic kidney disease, coronary artery disease, lower LVEF on admission (mean LVEF was 43 ± 11%), dyspnea at presentation [176], [177]. Also, it was shown that TTS might trigger rhythm disorders. Meanwhile, ventricular arrhythmias were the most common [178]. Rhythm disturbances mainly occurred in the first 48 h after admission to hospital, but asystole and AV-block were mainly out-hospital (before the hospitalisation). Cardiogenic shock and prolongation of the QT interval were identified as independent predictors of rhythm disturbances. The QT interval change could be explained by the role of increased late sodium current and decreased transient outward current (i.e., impaired depolarisation and repolarisation) [179] or by myocardial oedema (with QT prolongation 72 h after admission) [101]. Researchers have concluded that rhythm disturbances in TTS may indicate myocardial vulnerability in the acute phase so that patients with TTS and independent predictors of complications should probably be referred to the intensive care unit within 48 h of admission.

Moreover, atrial fibrillation is known to increase the risk of ventricular arrhythmias and cardiac arrest [180]. Therefore, such patients require longer hospital lengths of stay and careful monitoring after discharge.

In 2016 the retrospective study demonstrated paradoxical results of better intrahospital and long-term outcomes for patients with underlying diabetes mellitus who suffered from TTS [181]. However, in 2020 these results were not supported by Temidayo et al. It was demonstrated that TTS patients with DM had a higher risk for poor outcomes in a larger sample size [182]. At the same time, the 2021 research on “Diabetes Paradox” with a large Nationwide inpatient sample showed that TTS patients have a low prevalence of DM compared to patients without TTS. Moreover, complicated DM patients were found to have lower in-hospital mortality, probably secondary to blunted catecholamine mediated response caused by chronic hyperglycemia [183]. Therefore, further prospective research is warranted to evaluate the impact of DM on stress-induced cardiomyopathy.

## 13 Prevention

### 13.1 Pharmacological prevention

Until now, there has been no consensus regarding the pharmacological prevention of TTS. Thus, Santoro et al. found that the recurrence rate was lower with the use of beta-blockers (1.81%) compared with the control group (2.86%). The relapse rate with ACE inhibitors, aspirin, and statins was statistically less significant and amounted to 0.5% compared to 0% in the control group [136]. Contradicting results were obtained by Singh et al. in a *meta*-analysis showing that ACE inhibitors, and not beta-blockers, reduce the risk of recurrence [170]. Researchers suggested that β2-adrenoceptors are involved in the TTS pathogenesis so that the action of selective β1-adrenoblockers is neither practical nor helpful. In contrast, ACE inhibitors, according to the researchers, may reduce the risk of recurrent TTS through anti-inflammatory or sympathetic activity. The same results were obtained by Brunetti et al. in a 2016 study [131]. In 2021, a study was carried out to investigate the long-term effects of TTS. Patients were followed up for 5.2 years, and it was noted that recurrence and mortality were lower after therapy with beta blockers alone compared with patients taking ACE inhibitors only [172].

### 13.2 Taking antidepressants with caution

It is necessary to alleviate stress in everyday life to prevent TTS, and antidepressants should not be taken, especially SNRI, without prescription. Thus, obtaining proper medical history and identifying susceptible patients before starting antidepressants are vital in clinical practice. In addition, close follow-up care with a psychiatrist and a cardiologist is recommended to reduce TTS recurrences and long-term complications.

### 13.3 Changing lifestyle

As a preventive measure, it is advised to reduce the consumption of caffeine drinks and quit smoking since these are the risk factors that impair endothelial function and/or coronary blood flow reserve and increase adrenergic stimulation [184].

## 14 Conclusion

In summary, TTS is an increasingly recognized condition that can lead to less favourable outcomes comparable to those of acute coronary syndrome despite a self-limiting clinical course. Moreover, the two conditions can coexist, making diagnosis even more challenging. The existing knowledge about this syndrome is primarily based on consensus documents and current international registries. Therefore, controlled clinical trials and further research are needed to determine the optimal diagnostic approach, treatment, and prevention strategies.

## Declaration of Competing Interest

The authors declare that they have no known competing financial interests or personal relationships that could have appeared to influence the work reported in this paper.
